# The good, the bad and the ugly: Epigenetic mechanisms in glioblastoma

**DOI:** 10.1016/j.mam.2012.06.007

**Published:** 2013-07

**Authors:** Helena Carén, Steven M. Pollard, Stephan Beck

**Affiliations:** UCL Cancer Institute, University College London, 72 Huntley Street, London WC1E 6BT, United Kingdom

**Keywords:** Epigenetics, Glioma, DNA methylation, Central nervous system (CNS), Differentiation, Cancer stem cells

## Abstract

Cell type-specific patterns of gene expression reflect epigenetic changes imposed through a particular developmental lineage as well as those triggered by environmental cues within adult tissues. There is great interest in elucidating the molecular basis and functional importance of epigenetic mechanisms in both normal physiology and disease – particularly in cancer, where abnormal ‘-omic’ states are often observed. In this article we review recent progress in studies of epigenetic mechanisms in the most common primary adult brain cancer, glioblastoma multiforme. Three distinct areas are discussed. First, the evidence in support of ongoing ‘normal’ epigenetic processes associated with differentiation – as predicted by ‘cancer stem cell’ models of the disease. Second, identification of site-specific and global epigenetic abnormalities. Third, genetic disruptions directly within the core epigenetic machinery, exemplified by the recently identified mutations within isocitrate dehydrogenase genes IDH1/2 and variant histone genes H3.3/H3F3A. These constitute the ‘good, the bad and the ugly’ of epigenetic mechanisms in cancer.

## 1 Introduction

The term “epigenetics” was originally coined in the 1940s, when Conrad Waddington used it to refer to the study of processes by which genotypes give rise to phenotypes (Waddington, 1942). Nowadays, epigenetics is more commonly defined as a mitotic and/or meiotic heritable change in phenotype or gene expression caused by mechanisms other than changes in the underlying DNA sequence. DNA methylation, histone modifications and non-coding RNAs are the most intensively studied epigenetic mechanisms that affect gene expression. Disruption to each of these forms of regulatory control have been noted for a wide range of human cancers. For an excellent review of the history of cancer epigenetics the reader is referred to an article by Feinberg et al. (Feinberg and Tycko, 2004).

### 1.1 Epigenetic models of cancer development

Cancer initiation and progression involves a series of genetic alterations in a single founding cell that subsequently undergoes continuous evolution and clonal selection to generate a dominant clone – termed the *clonal genetic model of cancer* (Hanahan and Weinberg, 2000). In the early 1980’s it was discovered that changes to epigenetic state (DNA methylation) are also frequent in cancer, and can influence transcription of genes controlling growth-promoting and suppressing pathways (Feinberg and Tycko, 2004). As such there is a need to incorporate these events into current models of cancer. However, it remains unclear whether these alterations are necessary or sufficient for initiating and/or sustaining malignant cellular behaviour.

Epigenetic changes are often observed at the earliest stages of neoplasia within the altered tissue stem and progenitor cells. These observations have led to *the epigenetic progenitor model* (Feinberg et al., 2006). According to this model, transformation to a malignant state occurs in three steps. First, an essential early epigenetic disruption of stem/progenitor cells providing an expansion of an epigenetically permissive population. Second, an initiating genetic alteration in a ‘gatekeeper gene’ (tumour suppressor gene or an oncogene). Third, genetic and epigenetic plasticity resulting in an enhanced ability to stably evolve the phenotype. An important difference to the clonal genetic model is that the epigenetic ‘hits’ occur early, and are necessary to create an appropriate expansion of a polyclonal population, that is the cellular substrate for subsequent genetic alterations and transformation.

Subclonal genetic heterogeneity – so called *branching evolution models* – also creates heterogeneity within the tumour population. This has been described for acute myeloid leukaemia (AML) (Anderson et al., 2011) and more recently for brain cancers and renal carcinomas (Calabrese et al., 2007; Gerlinger et al., 2012). Programs of cellular differentiation, which are steered and stabilised by epigenetic mechanisms, also create phenotypic variation within the tumour cell population. Thus, tumours can display a variety of phenotypically distinct developmental/differentiation states layered on top of the genetic alterations. This intra-tumoural genetic and epigenetic heterogeneity likely underlies the inherent adaptability and resistance to therapies for most cancers. Establishing the validity of these models for each type of human cancer is critical. Likely a complicated picture will emerge, with each type of cancer as well as its various subtypes and stages of progression, displaying unique combinations and varieties of both genetic and epigenetic changes.

**Fig. 1:**
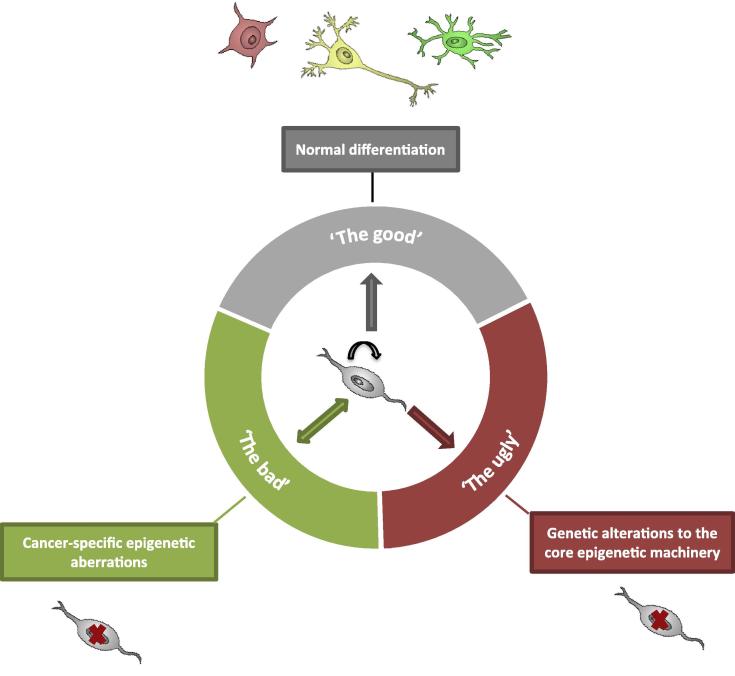
Three types of epigenetic mechanisms involved in differentiation of cancer stem cells. The ‘good’ mechanisms that operate to generate the cellular hierarchy in normal cell differentiation; the ‘bad’ epigenetic mechanisms, such as silencing of tumour suppressors, which are potentially reversible; and the ‘ugly’ mechanisms disrupted in a cancer irreversibly through genetic alterations to the core epigenetic machinery.

The specific focus of this article is the high grade glioma, glioblastoma multiforme (GBM). We review three major areas: (1) The ‘good’ epigenetic mechanisms that operate during differentiation to generate the cellular hierarchy. (2) The ‘bad’ epigenetic mechanisms, such as silencing of tumour suppressors, which are cancer-specific alterations that are nevertheless potentially reversible. (3) The ‘ugly’ mechanisms, disrupted in a cancer irreversibly through genetic alterations to the core epigenetic machinery (Fig. 1).

## 2 Glioblastoma

GBM is the most common and aggressive form of primary brain cancer. Current treatments for patients with these cancers are largely ineffective and the median survival is only fourteen months (Stupp et al., 2005). GBM is a form of a high-grade astrocytoma, with a large proportion of the tumour expressing the astrocyte marker, glial fibrillary acidic protein (GFAP) (Hamaya et al., 1985; Jacque et al., 1978). These tumours most commonly arise without any prior clinical history of a precursor neoplasia and are referred to as primary GBM. GBM can also develop over a period of 1–10 years from lower-grade glial tumours, either astrocytomas or oligodendrogliomas. These are then termed secondary GBMs. Although histologically similar, primary and secondary GBMs have different patterns of genetic alterations. A major advance in the field during the past few years has been the identification of mutations in *IDH*1/2 as a hallmark of secondary GBM. We will discuss the significance of this oncogene and its potential relevance to epigenetics later in this review.

Simultaneous genetic disruptions to the canonical P53, RTK/PI3K and RB/CDK pathways are common in primary GBMs (McLendon et al., 2008; Parsons et al., 2008). However, within primary GBM at least three or four different subtypes have emerged based on patterns of gene expression and genetic changes (Phillips et al., 2006; Verhaak et al., 2010). These subtypes have been termed ‘Proneural’, ‘Neural’, ‘Classical’ and ‘Mesenchymal’, based defining gene expression signatures. The precise biological significance and etiology of these ‘subtypes’ and whether they truly reflect distinct forms of the disease, or instead alternative stages of disease progression remains unclear. However, certain subtypes do correlate with distinct patient survival times and response to therapy (Phillips et al., 2006; Verhaak et al., 2010). The ‘proneural’ group has a high frequency of *TP*53 and *IDH*1 mutations as well as *PDGFRA* mutations and overexpression. It includes significantly younger patients and also correlated with increased survival compared to other subtypes. The ‘classical’ subtype is mainly characterised by a high frequency of *EGFR* aberrations and the absence of *TP*53 mutations, while the ‘mesenchymal’ subtype typically displays loss of the tumour suppressor gene *NF*1. The ‘neural’ group was less defined and did not show a unique deregulation of specific genes or pathways. This knowledge of the diversity of GBM will need to be incorporated into laboratory studies for a comprehensive understanding and modelling of the disease. Inevitably these differences need to be considered in design of new clinical trials – particularly targeted molecular therapies.

GBM is primarily a disease of adults. However, paediatric high grade glioma (HGG) has histological features of adult GBM and accounts for 15–20% of all childhood CNS tumours. Sadly only 10–30% of these patients survive more than 2 years after diagnosis. Recently our knowledge of the genetics of paediatric HGG has advanced considerably. Despite the similarities to GBM, studies have identified substantial differences in the molecular features underlying paediatric and adult high-grade gliomas and no correspondence to specific adult GBM subtype has been identified (Paugh et al., 2010). Common gain of chromosome 1q with a corresponding up-regulation in gene expression distinguishes childhood tumours from adult disease. *PDGFRA* is the most frequently occurring target of focal amplification in paediatric cases. Even those tumours that lack genetic amplification of *PDGFRA* or *PDGFRB* still show strong expression of the genes associated with *PDGFRA* amplification, suggesting a central role for this pathway. *IDH*1/2 mutations, commonly found in adult secondary gliomas, are also not present.

Noteworthy from these genetic analyses was the observation that paediatric GBM has a much more limited spectrum of genetic alterations than adult, with many of the tumours displaying a remarkably normal karyotype. This suggested the involvement of epigenetic disruptions, perhaps related to a specific spatial or temporal developmental context. In fact, in a highly significant advance, it has recently been reported by two groups that mutations within histone H3 and the associated chromatin modifying apparatus are frequent in paediatric brain cancers (discussed below) (Wu et al., 2012) (Schwartzentruber et al., 2012).

### 2.1 Cancer stem cell models of glioblastoma

Alongside the inter- and intra-tumoural genetic heterogeneity, there is also cellular heterogeneity within the GBM tumour population with regard to differentiation status, with some cells displaying immature stem/progenitor cell markers while others have more mature differentiated features. The cancer stem cell hypothesis proposes that this variety of cellular developmental states is reflective of the normal cellular hierarchy that would exist within that tissue. Consequently the tumour might be viewed as a complex tissue/organ, or a ‘caricature’ of normal development (Liang et al., 2002; Reya et al., 2001). Thus, the normal processes of differentiation may suppress some or all of the malignant hallmarks and force cells to enter a non-malignant, possibly terminally differentiated state. In that case, it is likely that epigenetic mechanisms are likely employed to abolish capacity for self-renewal and ‘lock in’ a differentiated state (Shackleton et al., 2009).

Within the adult central nervous system multipotent stem cells have been identified that generate neurons and glia through development and are retained in specific anatomical sites within the adult brain – namely, the hippocampus and walls of the forebrain ventricles (Ihrie and Alvarez-Buylla, 2011). Since these stem cells are long-lived compared to their differentiated progeny, it is intuitive that they may be susceptible to genetic and/or epigenetic mutations that result in tumourigenesis. Individual GBM tumours contain cells with neural stem cell characteristics, such as expression of CD133^+^ and nestin. Isolation and characterisation of this subpopulation using cell sorting for CD133 expression, or using other markers, has shown this subpopulationc tumour initiating (Singh et al., 2003). These putative GBM stem cells can be harvested and expanded *in vitro* using neural stem cell culture conditions where they remain tumour-initiating upon transplantation and have the capacity to differentiate (Singh et al., 2003; Galli et al., 2004; Lee et al., 2006; Pollard et al., 2009). As only a subpopulation of the tumour cells display long-term self-renewal and are tumour-initiating these studies lend support to a cancer stem cell, or hierarchical model of tumour growth. Such putative ‘cancer stem cells’ may arise through transformation of *bone fide* adult stem cells, committed precursors or via de-differentiation (Stiles and Rowitch, 2008), mouse models suggest each route may be possible (Lu et al., 2004) (Chow et al., 2011).

The relevance and generality of the cancer stem cell model as applied to solid tumours is an area of much debate (Visvader and Lindeman, 2008). GBM cells can respond to differentiation cues and alter patterns of gene expression – for example, response to BMP suppresses tumour initiation and is accompanied by the upregulation of GFAP (Piccirillo et al., 2006). However, to date there is little data regarding the stability of this differentiation. A challenge for the field is to confirm that those ‘normal’ or ‘good’ processes of lineage commitment and differentiation can indeed suppress malignant cellular behaviour, that this is then a stable and irreversible event, and finally to then identify the molecular basis of process. These issues will need to be addressed across the spectrum of subtypes and monitor at distinct phases of disease progression.

## 3 The variety of DNA methylation alterations in tumourigenesis

Silencing and activation of tumour suppressors and oncogenes can occur through changes to DNA methylation. While ‘bad’, these alterations are nevertheless potentially reversible and therefore represent potential therapeutic targets.

The methylation of cytosine in the CpG dinucleotide is a common modification of DNA in mammalian genomes. This reaction is catalysed by the enzymes DNA methyltransferases (DNMTs), which use S-adenosyl methionine (SAM) as the methyl donor. The majority of CpGs reside within repetitive elements that are methylated. Another site of variable methylation is within the CpG islands which are often associated with promoter regions of genes, and are usually unmethylated. DNA hypermethylation of CpG islands is associated with gene silencing and is also typically present at imprinted genes, repetitive elements and in genes on the inactivated X-chromosome in females (Reik et al., 1987; Wolf et al., 1984).

The first epigenetic disruption described in human primary tumours was gene specific hypomethylation (Feinberg and Vogelstein, 1983). Since then, DNA hypomethylation, both genome-wide, leading to chromosomal instability, and gene-specific, leading to oncogene activation, has been described as a general feature of tumours. Examples of genes targeted by DNA hypomethylation include R-ras in gastric cancer and cyclin D2 and maspin in pancreatic cancer (Akiyama et al., 2003; Nishigaki et al., 2005; Oshimo et al., 2003). Gene-specific hypermethylation was first described for the *RB* gene in retinoblastoma (Greger et al., 1989) and many other tumour suppressor genes have been reported as methylated in tumours, for example *p*16, *VHL*, *MLH*1, *APC* and E-cadherin (reviewed in (Jones and Baylin, 2002)).

The association between DNA methylation and tumourigenesis has been documented for many years but a formal proof that aberrant DNA methylation can directly lead to tumourigenesis came in 2011 (Teng et al., 2011). The tumour suppressor genes *RASSF*1*A* and *HIC*1 were subjected to targeted methylation in human mesenchymal stem cells (MSCs), which resulted in the formation of cancer stem cell-like cells and initiated transformation (Teng et al., 2011). By recruiting DNMTs to these loci, methylated DNA was introduced. The resulting cells showed genome-wide changes in DNA methylation and also altered p53 function. In addition, DNMT inhibitors were shown to reverse this phenotype.

Cancer-specific changes in patterns of DNA methylation that affect gene transcription have typically been ascribed to alterations at promoter regions that include CpG islands (Bird, 2002). However, as technologies for genome-wide analysis of DNA methylation patterns have improved it has now become clear that regions outside of the CpG islands may also have important regulatory roles (Irizarry et al., 2009). The differential methylation patterns that distinguish normal tissue types (T-DMRs; tissue differentially methylated regions) and patterns that can segregate colorectal cancer tissues from matched normal tissues (C-DMRs) are often located in CpG island ‘shores’ – regions with lower CpG density, surrounding the CpG islands (Irizarry et al., 2009). This was verified in another study using the Illumina GoldenGate technology to analyse 1505 CpG sites in 1628 samples of normal tissues, tumourigenic samples and non-cancerous disorders (Fernandez et al., 2012). Also in this study, CpG sites for which methylation discriminated between tissue types, were located in 5′ non-CpG-island regions. DNA methylation can also alter the expression of miRNAs, small non-coding RNAs that act as post-transcriptional regulators of gene expression (Saito and Jones, 2006).

Genome-wide DNA hypomethylation has been reported to occur in many tumour types, including GBMs (Cadieux et al., 2006). Hypomethylation of specific sequences was found to be associated with copy-number alterations of the adjacent euchromatin, suggesting that hypomethylation could predispose to specific genetic alterations that commonly occur in GBM. Many genes that are normally expressed in the healthy testis are activated by hypomethylation in cancer. One example is the melanoma-associated antigen 1 (*MAGE*-1), which has antigenic and immunotherapeutic value in GBM (Liu et al., 2004).

In GBM, there is frequent hypermethylation of tumour suppressors (*RB*1, *EMP*3, *RASSF*1*A* and *BLU*), cell cycle regulators (p16INK4a and p15INK4b), DNA repair genes (*MGMT*, *MLH*1), and genes involved in tumour invasion and apoptosis (*DAPK*, *TIMP*3 and *CDH*1) (Alaminos et al., 2005; Costello et al., 1994; Fukushima et al., 2005; Gonzalez-Gomez et al., 2003; Herman et al., 1996; Hesson et al., 2004; Horiguchi et al., 2003; Nakamura et al., 2001b; Uhlmann et al., 2003).

A well-studied example of a hypermethylated gene in GBM with important consequences for clinical management is *MGMT*, a DNA repair enzyme that is methylated in 68% of glioblastomas (Blanc et al., 2004). DNA hypermethylation of *MGMT* is more frequently associated with secondary GBMs than in primary GBMs or low-grade astrocytomas (Nakamura et al., 2001a). Silencing of *MGMT* is associated with an increased mutation rate and poor outcome in GBM. However, as GBMs with silenced *MGMT* cannot repair the DNA correctly, treatment with alkylating agents such as temozolomide has been demonstrated to be more effective in this group of tumours (Paz et al., 2004), making it a valuable biomarker for predicting drug responsiveness. Hence, *MGMT* methylation status is now being used in the clinical management of GBM patients.

In a study analysing 807 genes in 87 GBMs using Illumina GoldenGate methylation arrays, genes that were found to be hypermethylated in GBMs included many targets of the PRC2 (Polycomb repressive complex 2 (Martinez et al., 2009), suggesting that GBM might be driven by cells with stem cell-like epigenetic features. Seven genes (*HOXA*11, *CD*81, *PRKCDBP*, *TES*, *MEST*, *TNFRSF*10*A* and *FZD*9) were found hypermethylated in more than 50% of cases (Martinez et al., 2009). Of these, the gene *TES* has also previously been reported as methylated and down-regulated in GBM, as well as upregulated by pharmacological treatment with 5-aza-dC in GBM cell lines (Mueller et al., 2007).

### 3.1 ’New’ forms of methylation and its potential involvement in tumourigenesis

CpG methylation is the canonical form of DNA methylation in eukaryotes. However, methylation of other sites was noted many years ago (Ramsahoye et al., 2000; Salomon and Kaye, 1970; Woodcock et al., 1987). In 2009, non-CG methylation was reported to constitute around 25% of all methylation sites in embryonic stem (ES) cells (Lister et al., 2009). Increases in non-CpG methylation were found at gene bodies and depletion in protein binding sites and enhancers. Sites of non-CG methylation were removed following induced differentiation of the embryonic stem cells and conversely were restored in somatic cells reprogrammed to an ES-like state using induced pluripotent stem cell methodologies (Lister et al., 2009). Thus, it is possible that this specific mark is a unique feature of pluripotent stem cells. Whether the presence of non-CG methylation in pluripotent ES cells directly regulates differentiation potential or instead is a passive feature of the cells is currently unclear. In a recent study, however, non-CG methylation was also detected in the adult mouse frontal cortex, at the same average levels as in ES cells, suggesting that non-CG methylation is not a unique characteristic of pluripotent stem cells (Xie et al., 2012). A clearer picture will emerge once a broader range of tissue and tumour types has been assessed for this mark.

Another variant of DNA methylation that is of growing interest is 5-hydroxymethylcytosine (5hmC). 5hmC was originally identified in human and mouse brain and was proposed to have a role in epigenetic control of neuronal function (Kriaucionis and Heintz, 2009) (Tahiliani et al., 2009). This modification is catalysed through oxidisation of 5-methylcytosine by the ten-eleven translocation (TET) family. Three TET members have been characterised in vertebrates; TET1, TET2 and TET3, and their functions have been implicated in early embryogenesis, genome reprogramming, stem cell differentiation and haematopoiesis (Ficz et al., 2011; Gu et al., 2011; Quivoron et al., 2011). The discovery of 5hmC provided an important clue for the long postulated, but hitherto elusive process of active cytosine demethylation (Ooi and Bestor, 2008). In a series of elegant studies, 5hmC was shown to be further oxidised to 5-formylcytosine (5fC) and 5-carboxylcytosine (5caC) and the latter was found to be a substrate for Thymine DNA Glycosylase (TDG) that triggered excision of 5caC and replacement by unmodified cytosine by activation of the base excision repair (BER) pathway (He et al., 2011; Ito et al., 2011; Zhang et al., 2012).

Whether 5hmC has additional functions in addition to being an intermediate in DNA demethylation is unclear and remains controversial. According to one study, the distribution of 5hmC cannot be explained by the distribution of 5mC (Pastor et al., 2011). Genome-wide profiling found 5hmC to be enriched at exons and near transcriptional start sites, particularly those occupied with bivalent histone marks. In general, promoters marked with 5hmC were found to have lower levels of gene expression. This is contrasted by another study where 5hmC at promoters and CpG islands was found to be associated with increased transcription (Ficz et al., 2011). This study further found 5hmC to be largely absent in Dnmt triple knockout mouse ES cells, supporting the hypothesis that 5hmC is derived from pre-existing 5mC. Clearly, more work is required to clarify the possible functions of 5hmC, including the identification of 5hmC-binding proteins. The discovery of hydroyxymethyl pryrimidine (HMP) binding proteins in bacteria suggests that such proteins may exist in mammals as well (Devedjiev et al., 2004).

In a study analysing the tissue distribution of 5hmC, the highest 5hmC levels were observed in terminally differentiated cells, while less differentiated tissue stem/progenitor cells had very low levels (Haffner et al., 2011). Also, tumour samples from prostate, breast and colon had reduced levels compared to normal tissues. Immunohistochemistry and isotope-based liquid chromatography mass spectrometry (LC–MS) was used to investigate the presence and distribution of 5hmC in human brain and brain tumours (Kraus et al., 2012). The study showed a high level of 5hmC in the normal adult brain, immunohistochemistry identified 61.5% 5hmC positive cells in the cortex and 32.4% 5hmC in white matter areas. In tumours, the levels were much lower, from 1.1% in glioblastomas to 8.9% in grade I gliomas. Using LC–MS, the normal adult human brain tissues showed 0.7%–1.17% 5hmC/deoxyguanosine (dG) with lower levels in grade II diffuse astrocytomas, 0.24 % 5hmC/dG, and the lowest in glioblastomas, 0.078% 5hmC/dG. The levels of 5hmC were found to be unrelated to 5mC levels. This study suggests that the total amount of the 5hmC levels in tumours correlate with tumour differentiation stage. It will now be of interest to determine if 5hmC levels vary within the GBM population and ascertain whether this serves as an indicator of progression through the differentiation hierarchy or as a marker of terminal differentiation.

## 4 Disruption of the histone ‘code’

DNA is folded and compacted by histone and non-histone proteins into chromatin in eukaryotic cells. Chromatin is an important gene regulatory structure and variations are achieved through specialized histone proteins and by covalent modifications. Aberrations of histone modifications, the usage of histone variants as well as altered expression of chromatin-associated regulators are often disrupted in cancer. Histones can be modified post-translationally, which alters their interaction with DNA and nuclear proteins. Modifications of the histone tails (N-terminal regions that protrude from the nucleosome) include methylation, acetylation, phosphorylation, ubiquitination, sumoylation, citrullination and ADP-ribosylation (Kouzarides, 2007). It has been proposed that the combination of modifications constitute a code, the so-called “histone code”, which defines the status of the chromatin structure (Jenuwein and Allis, 2001).

It has become increasingly clear that chromatin modifications are at least as widespread and important as alterations in DNA methylation in cancers. Modifications such as the acetylation of lysine residues alter the charge and thus change the bulk of the nucleosome. This changes interactions with other nuclear components. Methylation, on the other hand, provides specific binding platforms for chromatin-associated proteins. H3K4 methylation positively regulates transcription by recruiting nucleosome remodelling enzymes and histone acetylases, while H3K27 regulates transcription negatively by promoting a compact chromatin structure (Ringrose and Paro, 2004; Santos-Rosa et al., 2003). The overproduction of specific histone methyltransferases are frequently observed in cancers, leading to aberrant methylation of lysine residues such as H3K4 and H3K27 (Hess, 2004). Moreover, at histone H4, the loss of acetylation at lysine 16 (H4K16) and the trimethylation of lysine 20 (H4K20) are frequently observed in cancer (Fraga et al., 2005). In a study of 230 gliomas using immunocytochemistry, the global expression of several histone modification marks was assessed (Liu et al., 2010). Based on WHO grade, histology, and the histone modifications H3K9Ac, H3K4Me2, H3K18Ac and H4K20Me3, 10 distinct prognostic groups were generated that were associated with significantly different progression-free and overall survival, suggesting that aberrant regulation of histone marks could also have a role in GBM.

The polycomb group (PcG) proteins form large complexes that are involved in gene silencing through modifications to chromatin. They are subdivided into two groups, the polycomb repressive complex 1 and 2 (PRC1 and PRC2). The histone modifications induced by the PRC1 and PRC2 complexes allow stable silencing of gene expression (Dellino et al., 2004). The trithorax group (TrxG) proteins have the opposite function to the PcG proteins and hence activate genes. TrxG proteins form multi-protein complexes that deposit histone marks that are associated to transcriptional activation. The complexes usually comprise of histone methyltransferases (HMTs) that contain SET-domains, which methylate lysine 4 of histone 3 (H3K4me3) (Schuettengruber et al., 2007).

The PcG proteins reversibly repress genes encoding transcription factors required for lineage choice and differentiation in stem cells (Ringrose and Paro, 2004). Interestingly, a significant fraction of genes that display DNA hypermethylation at promoters in GBM, are sites of repression by PRC2 in ES cells, an observation that has now been described for several solid tumours (Martin-Subero et al., 2009; Martinez et al., 2009; Ohm et al., 2007; Schlesinger et al., 2007). Acquisition of promoter DNA methylation at these repressed genes could ‘lock in’ stem cell phenotypes and initiate abnormal clonal expansion (Schuebel et al., 2006). Hence, genes targeted by PRC2 seem to be prone to acquire de novo DNA methylation during the development of GBM and this might link GBM pathogenesis to stem cells.

Altered activity of the polycomb group protein complexes, PRC1 and PRC2 is observed in GBM, through increased expression of PcG member EZH2 and this correlates with poor prognosis (Suva et al., 2009). Also, pharmacologic or genetic inhibition of EZH2 can prevent self-renewal and tumourigenicity of glioblastoma CSCs (Abdouh et al., 2009; Suva et al., 2009). *EZH*2-dependent epigenetic silencing of the BMP receptor 1B (*BMPR*1*B*) in a subset of glioblastoma tumour-initiating cells (TICs) has also been noted, thereby limiting the extent to which the TICs can respond to differentiation signals (Lee et al., 2008). *BMPR*1*B* was also shown to be down-regulated in about 20% of primary glioblastoma tumours, and this is correlated with increased promoter DNA methylation (Lee et al., 2008).

BMI-1 is a polycomb ring finger protein and oncogene that is part of PRC1. BMI-1 is not expressed in normal human astrocytes but is overexpressed in GBM tumours and highly enriched in CD133-expressing CSCs (Abdouh et al., 2009; Chatoo et al., 2009). It has been shown to be essential for tumour growth as it prevents the CD133^+^ cell population from undergoing apoptosis and/or differentiation. Down-regulation of BMI-1 in cultured GBM stem cells inhibited their growth and clonogenic potential (Abdouh et al., 2009). In addition, BMI-1 knockdown in mouse models suppresses malignant tumour formation, indicating a requirement for BMI-1 to sustain cancer stem cell renewal (Bruggeman et al., 2007).

The histone methyltransferase gene nuclear receptor SET domain containing protein-1 (*NSD*1) is another example of histone modifier that is silenced by DNA hypermethylation in glioma and neuroblastoma, (a cancer of the peripheral nervous system) (Berdasco et al., 2009). Restoration of NSD1 expression led to reduced colony formation and inhibition of cellular growth. The epigenetic inactivation of *NSD*1, in transformed cells, resulted in diminished trimethylation of H4K20 and H3K36. H4K20 methylation is associated with transcriptional silencing and H3K36 methylation is found primarily in active genes throughout the gene body (Bannister et al., 2005; Schotta et al., 2004).

## 5 The emerging links between genetics and epigenetics

In addition to reversible disruptions to DNA methylation and histone modifications, cancers may also accumulate genetic hits to the epigenetic machinery. These ‘ugly’ epigenetic mechanisms are permanent and irreversible as mutations are encoded within the genome. An example of a direct genetic disruption of the epigenetic machinery is the histone demethylase gene UTX which is mutated in several human cancers, including adult GBM (van Haaften et al., 2009). These inactivating UTX mutations leave UTX-regulated genes in an activated state, which might allow accelerated cell growth. Two recent findings have added to this type of epigenetic change. In 2008, activating point mutations in isocitrate dehydrogenase genes *IDH*1 and *IDH*2 were uncovered in secondary GBM following genome-wide sequencing (Parsons et al., 2008). More recently, mutations in histone H3 and associated regulatory complexes have been identified in paediatric HGG (Wu et al., 2012) (Schwartzentruber et al., 2012).

### 5.1 IDH1/2 mutation and DNA methylation

The discovery of *IDH*1/2 mutations in GBM was unexpected. However, similar mutations have now been described in acute myeloid leukaemia (AML) (Figueroa et al., 2010), chondrosarcoma and in Ollier disease and Maffucci syndrome (Amary et al., 2011). Initially these changes were thought to primarily operate through a disruption to cancer metabolic pathways. However, the changes in the IDH1 protein result in aberrant production of 2-hydroxyglutarate (2-HG), an ‘oncometabolite’ which inhibits TET2 enzymatic activity and leads to a hypermethylation promoter phenotype (Figueroa et al., 2010). Thus, an unexpected link between cancer cell metabolism and epigenetic state is emerging. GBMs harbouring an *IDH*1 mutation differ not only in their genomic and epigenomic profiles, but also in their demographic, anatomic, and phenotypic presentation and follow a different clinical course. This supports the notion that these types of glioma may be viewed as a quite separate form of disease with distinct aetiology (Lai et al., 2011).

*IDH*1/2 mutated gliomas show distinct epigenetic profiles, characterised by a high frequency of methylated CpG islands, the “CpG island methylator phenotype” (CIMP) (Watanabe et al., 2009). The CIMP profile is characterised by a large numbers of methylated CpG islands in a tumour. CIMP was first described in colorectal cancers where two distinct subgroups of tumours were identified that harboured low and high levels of tumour-specific DNA methylation, respectively (Toyota et al., 1999a). CIMPs have since then been described also in gastric-, lung-, liver-, pancreatic-, oesophageal- and ovarian cancer, leukaemias and gliomas (Noushmehr et al., 2010; Roman-Gomez et al., 2005; Shen et al., 2002; Strathdee et al., 2001; Suzuki et al., 2006; Toyota et al., 1999a,b; Ueki et al., 2000). Despite this, there is still no unifying definition of genes included in the CIMP in the respective tumour types.

The functional consequences of *IDH*1 mutations and a role in the establishment of the glioma-CIMP (G-CIMP) has been explored using immortalised primary human astrocytes and isogenic cells expressing either mutant or wild-type *IDH*1 (Turcan et al., 2012). Expression of mutant, but not wild-type *IDH*1 in human astrocytes, resulted in the production of 2-HG and the establishment of CIMP by remodelling of the methylome and dramatic effects on the transcriptome suggesting these mutations are primary drivers of the epigenetic changes. G-CIMP was also shown to be highly dependent on a mutation in *IDH*1 or *IDH*2; 99% of the tumours with a G-CIMP possessed a mutation in either of these genes and none of the tumours without a mutation was G-CIMP positive. Further, it was shown that the G-CIMP is a significantly better predictor of patient survival than *MGMT* methylation or mRNA expression (Turcan et al., 2012). 2-HG can also inhibit histone demethylation and this can be sufficient to block the differentiation of non-transformed cells (Lu et al., 2012).

Supervised analysis of gene expression data found a statistically enriched gene signature in *IDH*-mutant samples that was independent of tumour grade and recurrence status. The top identified functional categories included regulation of astrocyte and glial differentiation, suggesting a role in cell differentiation. When ectopically expressing wild-type or mutant IDH1 or IDH2 in 293T cells the authors found an increase in histone methylation compared to the wild-type IDH1/IDH2 and this always correlated with the intracellular 2-HG levels. When comparing tumours with mutant versus wild-type IDH, there is a significant increase of the repressive histone mark H3K9me3 in the latter. To explore the function in CNS cells, immortalised normal human astrocytes were transduced with either wild-type IDH1 or R132H mutant IDH1. Compared to parental cells, late-passage cells expressing mutant IDH exhibited elevated levels of histone methylation marks. The total levels of CpG methylation was also shown to increase in IDH mutant cells, consistent to the G-CIMP seen in IDH mutant gliomas.

Together these studies have unexpectedly revealed a link between what was initially thought to be a change in cancer metabolism, to a direct influence on gene expression through inhibition of TET2. It is now crucial to determine whether the effects of mutant IDH can be suppressed. The elevated 2-HG levels, which are characteristic of IDH1 and IDH2 mutated tumours, is also potentially useful as a biomarker to identify *IDH*1/*IDH*2 mutational status or monitor tumour growth or treatment efficacy in a non-invasive way. However, it is not yet known if elevated levels from glial tumours can readily pass through the blood–brain barrier and be detected in blood or urine.

### 5.2 Histone H3 mutations in gliomas

Excitingly, two recent studies have uncovered a direct genetic disruption at genes encoding histone proteins and chromatin remodelling genes in paediatric high grade gliomas and diffuse intrinsic pontine gliomas (DIPGs) (Wu et al., 2012) (Schwartzentruber et al., 2012). The Wu et al. study, identified in DIPG and non-brainstem paediatric glioblastomas (non-BS-PGs), mutations in the genes *H*3*F*3*A* (encoding histone H3.3), or in the related *HIST*1*H*3*B* (encoding histone H3.1). These were observed in 78% of DIPGs and in 22% of non-BS-PGs respectively (Wu et al., 2012).

The Schwartzentruber et al., study also identified mutations in H3.3, and in chromatin remodelling genes (Schwartzentruber et al., 2012). Somatic mutations in the H3.3-ATRX-DAXX chromatin remodelling pathway were identified in 44% of the paediatric glioblastoma tumours. Mutations in the *H*3*F*3*A* gene, which encodes for H3.3, were highly specific to GBM cases and were observed in 31% of tumours, leading to amino acid substitutions at two critical positions within the histone tail (K27M and G34R/G4V). Mutations in the genes *DAXX* and *ATRX*, which encodes for two subunits of a chromatin remodelling complex required for H3.3 incorporation at pericentric heterochromatin and telomeres, were identified in 31% of samples overall and in 100% of the tumours with a H3.3 mutation. The mutations in *H*3.3, *ATRX* and *DAXX* were shown to be largely specific to paediatric cases, further highlighting the distinct molecular pathology of these cancers.

These mutations lead to a p.Lys27Met amino acid substitution. In addition, 14% of the non-BS-PGs had somatic mutations in *H*3*F*3*A* causing a p.Gly34Arg alteration. The alterations are both located in key regulatory sites within the histone tails that are normally associated with gene repression or gene activation depending on the histone tail modification present. Trimethylation of Lys27 in histone H3 (H3K27me3) is associated with the silencing of genes and monomethylation (H3K27me) is associated with gene activation. Acetylated Lys27 has also been shown to be associated with transcriptionally active regions. The replacement of Lys27 with a methionine suggests a loss of function, as it removes the ability to methylate or acetylate this position. However, the mutations are present in the heterozygous state and always encode the same amino acid substitutions, which suggest a gain-of-function phenotype. Thus, the functional consequences of these alterations still remain to be elucidated. Whether these genetically-driven direct disruptions to epigenetic mechanisms in paediatric glioma are mirroring changes in adult GBM that are driven by alternative mechanisms remains to be determined.

## 6 Epigenetic therapy

Epigenetic pathways are important therapeutic targets. The altered ‘bad’ epigenetic defects that accumulate in cancer are potentially reversible, and the ‘good’ epigenetic mechanisms which may still operate in cancer stem cell driven contexts could be promoted through inductive differentiation promoting signals. However, the ‘ugly’ epigenetics driven directly through altered genetics within key regulatory molecules may be more challenging to suppress.

Treating tumours with demethylating agents or histone deacetylases could activate silenced tumour suppressor genes and have clinical value. More than 40 years ago, the cytidine ribose nucleoside analogue 5-azacytidine was discovered as a potent agent for cancer treatment (Sorm et al., 1964). It was also subsequently shown to be an inhibitor of DNMT. In the cell, 5-azacytidine is modified to deoxyribonucleoside triphosphate and is incorporated into DNA where it is methylated by DNMT. DNMT is unable to dissociate from the methylated base and the methyltransferase activity in the cell thereby rapidly diminishes during replication. 5-aza-2′-deoxycytidine (decitabine) and zebularine are other examples of nucleoside analogues (Zhou et al., 2002). 5-azacytidine and 5-aza-2′-deoxycytidine have both been approved by the FDA for the treatment of myelodysplastic syndrome (Watanabe et al., 2011). However, these compounds rapidly degrade in the body. Zebularine is another demethylating agent which is more stable and can be administered orally (Marquez et al., 2005).

The fact that the nucleoside analogues need to be incorporated into DNA during DNA synthesis limits the activity of the drugs in slowly proliferating cells, potentially limiting their effectiveness against quiescent cancer stem cell populations. Non-nucleoside DNMT inhibitors are therefore under development, also with a second aim of avoiding the toxicity associated with the incorporation of nucleoside analogues into DNA. The future also holds great promise for targeted epigenetic reprogramming through the use of technologies such as engineered zinc finger nucleases (ZFNs), artificial transcription factors (ATFs) and transcription activator-like effector nucleases (TALENs) (Wood et al., 2011). Successes in this direction include targeted DNA methylation in cancer cells using DNMT3A coupled to zinc-finger ATFs (Rivenbark et al., 2012) and targeted demethylation by fusing thymine DNA glycosylase (TDG) to a sequence-specific DNA binding domain (Gregory et al., 2012).

Histone deacetylases (HDACs) are a family of 18 deacetylating enzymes that remove acetyl groups from lysine residues of histone proteins, as well as on other proteins including transcription factors (Witt et al., 2009). HDACs are grouped into four classes among which classes I, II and IV are called “classical” HDACs. This group of HDACs can be inhibited by small molecule compounds called HDAC inhibitors (HDACi). Class III HDACs are called sirtuins and differ from classical HDACs in their catalytic mechanism and co-factor requirements. HDACs regulate the conformation and activity of chromatin through their deacetylation of the histone proteins H2A, H2B, H3 and H4. The interaction between positively charged histones and negatively charged DNA is thus controlled. HDACs mostly act as part of large multi-protein complexes that function as transcriptional co-repressors. Euchromatic regions with active transcription are associated with low HDAC activity, whereas condensed, transcriptionally inactive heterochromatic regions have high HDAC activity.

HDAC inhibitors affect histone acetylation but also facilitate replication-independent DNA demethylation and can therefore be utilised to induce demethylation in post-mitotic non-dividing tissues and in slowly proliferating cells (Cervoni and Szyf, 2001). HDACi can synergize with DNA damaging agents in arresting/killing glioma cells *in vitro*, which might be due to that HDACi promote a more open chromatin conformation and therefore permit better access of DNA damaging agents to the chromatin, with an increased efficacy of these agents. The HDACi SAHA (Vorinostat) has been successfully utilised in clinical trials of patients with cutaneous T cell lymphoma (Duvic et al., 2007). SAHA can induce cell cycle arrest, terminal differentiation and cell death in transformed cells. Several studies have shown that it has activity against gliomas (Eyupoglu et al., 2005; Ugur et al., 2007). A phase II trial of Vorinostat showed that Vorinostat monotherapy is well tolerated in patients with recurrent glioblastoma but has modest single-agent activity (Galanis et al., 2009). Immunohistochemical analysis showed an increase in acetylation of histones H2B, H3 and H4 in a majority of the patients after treatment and gene expression showed changes in genes regulated by Vorinostat, such as upregulation of E-cadherin. Trials with combinatorial treatments are warranted to elucidate if HDACi can be beneficial to glioblastoma treatment. However, in a recent trial combining Vorinostat and the proteasome inhibitor bortezomib in patients with recurrent glioblastoma, this combination was shown to be clinically ineffective (Friday et al., 2012).

## 7 Concluding remarks

**Fig. 2:**
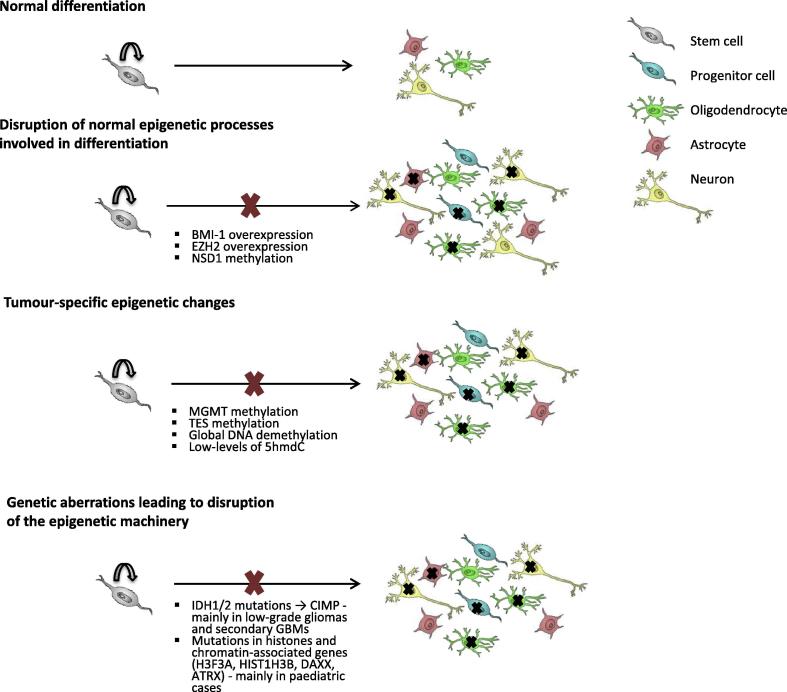
Multiple routes to altered epigenetics within GBM cells. Disruption of mechanisms that generate cellular hierarchy in normal cell differentiation, aberrant DNA methylation – gene specific and global, and direct genetic hits to the core epigenetic machinery can all occur in GBM.

Exploration of epigenetic processes within GBMs is clearly of great importance for a full understanding of the biology of this disease, and for the management of patients (Fig. 2). As described in this review, disruption of mechanisms that generate the cellular hierarchy in normal cell differentiation, aberrant DNA methylation of tumour suppressor genes and oncogenes and direct genetic hits to the core epigenetic machinery can all occur in GBM. Several interesting insights have been made during the past decade. Although histologically similar, paediatric differs from adult forms of the disease. Even for adult there are clear differences between primary GBM and secondary GBM, and further subclassification into three or four major subtypes. Each of these variants has shared pathological and histological appearance, but quite distinct different spectrum of molecular and cellular abnormalities. Thus, we are dealing with a very heterogeneous group of diseases. Based on the steady progress of various international efforts such as the International Cancer Genome Consortium (www.icgc.org) and Cancer Genome Atlas (cancergenome.nih.gov), we should soon have comprehensive reference data defining the spectrum of all major ‘omic’ changes for a range of gliomas and related malignancies. These will provide a foundation for exploring the functional consequence and cell type specific effects of various altered pathways.

Intra-tumoural heterogeneity of cell populations also presents a major obstacle to our understanding of GBM. It is therefore now important to make use of single cell analysis and try to reconcile cancer stem cell models of the disease with clonal genetic evolution models. The recent findings of somatic mutations in key regulatory genes (*H*3.3, *ATRX* and *DAXX*) highlight the importance epigenetic alterations have in the development of glioma. Efforts to understand the biological significance of these various disruptions, particularly the elucidation of which mechanisms are key functional players either early during tumour initiation, during disease progression or in response to therapies, will help in design of rational therapeutic strategies and improve currently dismal patient outcomes.
